# Understanding the needs of postpartum emerging adults with substance use disorders to improve recovery supports

**DOI:** 10.3389/fpubh.2025.1521093

**Published:** 2025-10-29

**Authors:** Camille C. Cioffi, Lauren E. Lewis, Jeff Gau

**Affiliations:** ^1^Prevention Science Institute, University of Oregon, Eugene, OR, United States; ^2^University of Connecticut School of Medicine, Farmington, CT, United States

**Keywords:** postpartum, emerging adults, substance use disorders, needs assessment, barriers

## Abstract

**Introduction:**

Postpartum is a critical time point to support recovery among emerging adults with substance use disorders (SUD), however, support for postpartum emerging adults with SUD is insufficient. The goal of the current study was to identify key intervention objectives to improve recovery services for postpartum emerging adults with SUD.

**Methods:**

We conducted a needs assessment with 100 women with past or present SUD. We asked women to report on their postpartum experiences retrospectively for post-pregnancy experiences between the ages of 18–29. Participants provided information on their pregnancy experiences and challenges. They also provided responses to open-ended questions to inform solutions to address challenges.

**Results:**

The top challenges for postpartum emerging adults included anxiety and depressive symptoms, economic resources, and lack of intimate and supportive social relationships. Participants described solutions to destigmatize societal perceptions of postpartum emerging adults with SUD, provide mental health services that are responsive to social challenges, provide practical economic assistance, improve awareness of services, and support the formation of informal support networks.

**Discussion:**

This study provides insights on the needs of postpartum emerging adults. These data are actionable for both researchers and practitioners to inform recovery services improvements.

## Introduction

Pregnancy is a natural period for substance use cessation and concentrated societal efforts to prevent fetal substance exposure (1–5). While many pregnant people abstain from substance use, some may return to use during the postpartum period (i.e., the 12 weeks to 1 year post birth) (6, 7). There have been multiple recent calls to action to identify and support postpartum people with substance use disorders (SUD) as it is a period of opportunity for recovery and vulnerability to return to use for people with SUD (8–10). Emerging adults (ages 18–29) may be particularly vulnerable in the postpartum period. Broadly, postpartum includes physical, social, and psychological changes as a function of hormonal fluctuations, recovery from childbirth, the physical demands of caring for an infant (e.g., feeding, sleep disruptions) and/or the demands of emotionally and physically processing perinatal loss and changing relationship with a partner or spouse or the demands of singleness as it relates to both parenting and loss experiences (11–13). For emerging adults, added difficulties may include adjusting to a new identity as a parent, disruptions in social relationship with non-peer parents, education disruptions, greater needs for social support, and heightened stigmatization in day-to-day interactions including interactions with healthcare professionals (14). In general, emerging adults often experience treatment dropout rates at a higher percentage than that of their adult peers (15, 16), thus, SUD and recovery introduce even greater complexities.

### Recovery services for postpartum emerging adults with SUD

A nationally representative survey of adults in the United States (*N* = 1,899) found that 40% of people who reported being in recovery entered into it during their emerging adult years, most of whom, were women (17). Though individual definitions may vary, recovery generally is not simply defined by abstinence from substances but can be defined as an improved sense of stability across all facets of life (18, 19). Thus, recovery services include housing, social services, peer support, and case management spanning a variety of domains aimed at addressing structural, interpersonal, and individual needs (20, 21). Within these domains, there are barriers that present challenges for SUD recovery during the postpartum period (22–28). However, extant research is limited by the number of qualitative reports, and the various geographical locations including research from Australia and Norway, which reduce the generalizability of findings to a U.S. sample. Nevertheless, these prior studies suggest that structural barriers place burden on postpartum emerging adults by making it more difficult to access treatment including structural racism and socioeconomic factors such as insurance coverage, housing status, and access to transportation (29–31). Structurally disadvantaged family composition, lack of stable housing, inequitable health care access, and general stigma around SUDs also create barriers to access (32–36). Shortcomings of the current medical environment (e.g., lack of integrated SUD treatment services, multiple caregivers, limited case management support, barriers to basic needs services) can result in fragmented care and lack of access to needed recovery, medical, social, and basic needs supports (37–42). Decisions to engage in or maintain recovery are complicated further by the potential of child welfare involvement (43–47). For those who have the opportunity to parent, they may experience fear of child welfare involvement and as a result may be less likely to engage in treatment services (3, 29).

Interpersonal barriers limit the ways postpartum emerging adults with SUD feel comfortable interacting with others (48–50). Lack of social support makes recovery, pregnancy, and parenting notably more challenging (4, 26, 27, 30, 36, 48, 50–55). Additionally, postpartum emerging adults may experience discrimination based on multiple aspects of identity, including substance use leading to disenfranchisement and disengagement with conventional care (4, 48, 56–58). Some individuals may feel coerced into recovery processes before they are ready or may be unsure of whether to maintain abstinence following pregnancy (7, 59). Loss experiences are more stigmatized for emerging adults with SUD with societal communication placing blame and expressing harmful sentiments that insinuate or directly state that the child welfare involved or bereaved parent are not meant to be a parent in the first place (60, 61). Child removal and perinatal loss may decrease the likelihood of treatment utilization. In addition, as a result of healthcare-related stigmatization, postpartum emerging adults with SUD experience a lack of clarity of what will happen during care visits, lack of clarity of what infant withdrawal will look like, discrimination in the hospital, challenges finding treatment centers who can accommodate them and their baby, and overall lack of choice (36). This stigma prevents people from seeking and using needed services including SUD treatment (4, 36, 48, 51, 52).

Individual barriers include the circumstances and individual characteristics or experiences that may interfere with finding and engaging with treatment services. For example, caring for a baby with Neonatal Abstinence Syndrome (62), having children who are placed in custody of child welfare due to issues related to parent SUD or other reasons (63, 64), and perinatal loss experiences are all unique experiences with their own set of considerations. For example, both child removal and perinatal loss have been associated with increases in psychological distress (43, 65) and thus may increase a person's likelihood of continuing or resuming frequent use after a period of abstinence or improvement. Conversely, retaining custody protects against parent return to use (66). Distressing and traumatic experiences, including those caused by the aforementioned punitive systems, may hinder timely treatment utilization (44, 67–70). Additionally, mental health and health considerations may make treatment utilization more complicated such as the presence of chronic diseases, infections, psychiatric disorders, and polysubstance use (53, 54, 71). Emerging adulthood is also marked by identity formation postpartum experiences may make it more complicated to engage in this developmental process (72). However, the natural identity shifts experienced while becoming a parent, and emerging adulthood may facilitate reductions in substance use for subpopulations of individuals with SUD as a tipping point to enhance readiness to change (68). Prior research on reasons for low treatment utilization for emerging adults has not included an understanding of postpartum contributions (73). While these challenges have been identified, primarily by qualitative extant literature, the current state of interventions prioritize approaches to improve individual behavioral motivation for treatment utilization and lacks focus on key barriers and facilitators specific to emerging adults (74). Quantitative research is needed to understand the priorities of postpartum emerging adults to improve recovery service.

Support for postpartum emerging adults with SUDs is currently insufficient (75). To our knowledge there are no tailored interventions for postpartum emerging adults with SUD to improve recovery services. This leads to missed opportunities to support this population during a critical transition point when they are establishing their identity both as an adult and a parent, regardless of the pregnancy outcome (14). Without focused, non-judgmental, comprehensive support, return to use becomes more likely (7) and they are at greater risk for recurrence of SUD (44, 76, 77). In summary, there are a variety of important factors to address to improve the recovery services among postpartum emerging adults with SUD. The current study sought to identify service gaps to prioritize intervention.

### Current study

The purpose of our study was to assess the needs of emerging adults experience during the postpartum period to inform intervention research and services. We sought to describe pregnancy experiences among postpartum emerging adults with SUD and characterize structural, interpersonal, and individual barriers to generate a list of priorities for intervention. We also sought to understand whether there were differences in priorities for the most common postpartum experiences: navigating pregnancy and infant loss and caring for an infant. We characterize discrimination experienced by postpartum emerging adults to clarify the intersectional ways that stigmatization is experienced for consideration in interpersonal and structural interventions. Finally, we had the goal of providing narrative information from the perspectives of the women impacted by these experiences on ways society can improve support for postpartum emerging adults and individual resilience. The findings from our study could be used to determine how to improve recovery services for postpartum emerging adults.

## Methods

### Settings, participants, and procedures

We leveraged two existing research databases to conduct our research: The Center on Parenting and Opioids Data Collective and Research Match. Research Match is a volunteer database where volunteers self-report medical conditions including SUD diagnosis. For Research Match, we limited our participant pool to individuals who had a live birth or miscarriage, and a diagnosis of SUD. More than 90% of participants were recruited from the Data Collective. The Data Collective recruits participants in-person from SUD treatment and recovery services (e.g., outpatient SUD treatment, opioid treatment programs, recovery community organizations). At the time of the study Data Collective only included pregnant, recently pregnant, and parenting people with SUD in the database. We provided institutional review board approved text messages and email templates to these two recruitment platforms to send to participants who were likely to meet our eligibility criteria. Recruitment messages (text and email) were sent to all people whose sex assigned at birth was female. Our message invited potential participants to take part in a one-time 30-min survey. Participants were offered a $30 gift card to incentivize participation. Those interested completed a brief screening survey to assess eligibility. Participants were eligible if they had ever gone to treatment for SUD (implying a past SUD diagnosis by a professional) or self-reported binge drinking or use of at least one non-nicotine substances at least weekly in the past year using the NIDA Quick Screen Assist (78). They also had to report having been pregnant between the ages of 18–29. The study project coordinator verified eligibility by reviewing the screening information and engaging with the participant via text or email prior to distributing the survey. Additionally, following survey completion, the study coordinator verified data for accuracy and completion and followed up with participants in instances of missing data to obtain a more complete and accurate record. The total sample for analysis was 100 participants.

### Measures

Our self-report, retrospective survey included questions on participant demographic characteristics, pregnancy experiences, substance use during pregnancy and postpartum, postpartum challenges, and discrimination. Our survey also included open-ended items to further assess challenges, solutions, and strengths of women in recovery with SUD during challenging postpartum experiences.

#### Demographics

Demographics assessed participant race and ethnicity, gender identity, age, and highest level of education completed. They also assessed whether they identified as houseless or in recovery during some or all of the time after their pregnancies from 18 to 29.

#### Pregnancy experiences

We assessed pregnancy experiences by asking participants how many times they had been pregnant between the ages of 11–17, 18–29, and 30–39. Participants were then asked to report, of those pregnancies, how many resulted in the following: *live birth and retained child custody, live birth with child removal, miscarriage (spontaneous abortion), termination (therapeutic abortion), or other loss (e.g., neonatal death, stillbirth, medical anomaly)*.

#### Substance use during pregnancy and postpartum

We assessed substance use during pregnancy and postpartum by asking participants first whether they used substances during pregnancy and in the year after pregnancy within each age group (i.e., 11–17, 18–29, and 30–39). Participants who indicated substance use during pregnancy or postpartum were then asked to select which substances they used during and after pregnancy. They could select all that apply from the following response options as informed by the NIDA Quick Screen (78): *alcohol, nicotine/tobacco, cannabis (marijuana, pot, grass, hash, etc.), cocaine (coke, crack, etc.), prescription stimulants not as prescribed (Ritalin, Concerta, Dexedrine, Adderall, diet pills, etc.), methamphetamine (speed, crystal meth, ice, etc.), inhalants (nitrous oxide, glue, gas, paint thinner, etc.), sedatives or sleeping pills (Valium, Serepax, Ativan, Xanax, Librium, Rohypnol, GHB, etc.), hallucinogens (LSD, acid, mushrooms, PCP, Special K, ecstasy, etc.), street opioids (heroin, opium, etc.), prescription opioids not as prescribed (fentanyl, oxycodone [OxyContin, Percocet], hydrocodone [Vicodin], methadone, buprenorphine, etc.), other*. They were also asked to report on the frequency of use for each of these substances.

#### Postpartum challenges

To assess challenges during the postpartum period, participants were prompted: “Please answer the following questions about your experiences in the first year after your pregnancy or pregnancies when you were ages 18–29, especially during the times when you felt like you needed more support. Did you experience challenges with the following?” Participants were provided with a list of potential challenges. Challenges were informed by extant qualitative literature (4, 30, 52) and derived using a bioecological perspective (79) to consider individual (e.g., *understanding information provided by healthcare providers; concentrating, remembering, or making decisions*), interpersonal (e.g., *having someone to talk to about major life decisions; finding safety from people who insulted, talk down to you, or screamed or cursed at you; because of my substance use, my medical providers were unkind to me*), societal (e.g., *access to education like job training, high school diploma, GED or equivalent*; *accessing 12-step, SMART recovery, or other community-led support groups*), and environmental challenges [e.g., *finding a place to live where your health was supported (e.g., good air quality, no mold, no lead paint)*]. There was a total of 62 challenges. For each challenge, a participant indicated their response on a three-point scale: *Yes, major challenge*; *Yes, minor challenge*; *Not a challenge*. The 10 challenges with the highest percentage endorsement of *Yes, a major challenge*, were summarized for this report.

#### Discrimination

For discrimination, participants were asked to respond to the question: “What are some of the main reasons you have experienced discrimination?” Potential responses included: *your ancestry or national origins, your gender, your race, your age, your religion, your height, your weight, some other aspect of your physical appearance, your sexual orientation, your education or income level, your housing status, a physical disability, your shade of skin color, your affiliation with a tribe, the language you spoke or your accent, your substance use, your income or social class, other, or I don't think I've experienced discrimination*.

### Open-ended questions

Participants were asked to select up to their top five major challenges. For each of the challenges they selected, they were asked to clarify, “what prevented you from getting the access you needed to each of the following needs?” and “what could other people or society have done to help?” At the end of the survey, participants were asked to reflect on “what is something positive that happened during that time in your life or something that you are proud of?” The purpose of this question was to uplift individual strengths and hope for positive change in the lives of an otherwise stigmatized population, and to end the survey with an opportunity for positive reflection.

### Analyses

Descriptive statistics were used to summarize the quantitative participant responses, including means, standard deviations, and frequencies. Text data were summarized by the first author by identifying emergent themes and tagging each narrative with associated themes. After tagging each narrative, a summary for each question was generated and quotes were selected with the goal of supporting the claims made in the summary statements.

## Results

### Participant characteristics

Participant demographic characteristics are provided in Table 1. On average, participants were 34.7 (*SD* = 6.7) years old, and all identified as women. Most respondents were White (93% White only) and their highest level of education was a GED, high school diploma, or some college (62%). Nearly all participants identified as being in recovery from SUD at the time of completing the survey (96%). However, 57% also reported that they used drugs including heroin, methamphetamine, cocaine, or fentanyl in the past year.

**Table 1:** Sample demographic characteristics of women with past or present substance use disorder, reporting on their postpartum experiences as an emerging adult for the present study (*n* = 100).

**Demographics**	***n***	***%***
**Race (select all that apply)**		
American Indian or Alaskan Native	4	4.0
Asian	1	1.0
Black	2	2.0
Pacific Islander	1	1.0
White	93	93.0
Latina, Latino, Latinx, or Hispanic	8	8.0
**Highest level of education**		
Some middle school/junior high	1	1.0
Junior high school/middle school (8^th^ grade)	5	5.0
Some high school	16	16.0
GED or high school diploma	30	30.0
Some college or vocational training	31	31.0
2-year degree	10	10.0
4-year degree	5	5.0
Graduate school or other post-secondary education	2	2.0
In recovery during postpartum (% yes)	77	78.0
In recovery at the time of the study (% yes)	96	96.0
Past year elevated alcohol use (4+ drinks per day)	41	41.0
Past year tobacco use	88	88.0
Past year prescription drug misuse	36	36.0
Past year other drug use (e.g., heroin, meth, cocaine, fentanyl)	57	57.0

The amount of missing data for the 11–17 pregnancy items ranged from 4 to 14% (1–3 participants), for 18–29 years 4–19% (4–19 participants) and for 30–39 years 11–26% (4–10 participants). The percentages reported in this table are for observed data.

Participant pregnancy experiences are summarized in Table 2. In line with our eligibility criteria, all participants had at least one pregnancy experience between the ages of 18–29. On average, participants were pregnant 2–3 times from the ages of 18–29 (*M* = 2.5, *SD* = 1.5, *range* = 1–9). During this age, 78% reported at least one live birth and retained custody, 16% reported at least one live birth that resulted in child removal, 32% reported having a spontaneous abortion (miscarriage), 21% reported having a therapeutic abortion, and 5% reported having another loss experience. These are likely underestimates as around 15% of respondents did not respond to questions about removal and loss.

**Table 2:** Pregnancy experiences among women ages 18–29 with past or current substance use disorders (*n* = 100).

**Pregnancy experiences**	***n***	***%***
**Number of pregnancies**		
One	30	30.0
Two	29	29.0
Three	20	20.0
Four	14	12.0
Five	5	5.0
Six or more	3	3.0
^a^ **Had a live birth, retained child custody**		
No	18	18.0
Yes	78	78.0
Did not respond	4	4.0
^a^ **Had a live birth, child removal**		
No	69	69.0
Yes	16	16.0
Did not respond	15	15.0
^a^ **Had a miscarriage (spontaneous abortion)**		
No	54	54.0
Yes	32	32.0
Did not respond	14	14.0
^a^ **Had an abortion (therapeutic abortion)**		
No	64	64.0
Yes	21	21.0
Did not respond	15	15.0
^a^ **Had another loss**		
No	76	76.0
Yes	5	5.0
Did not respond	19	19.0

^a^Among women who indicated they had a pregnancy.

Participant substance use during pregnancy is presented in Table 3. Most women reported using substances during at least one of their pregnancies between ages 18–29 (75%) and in the year after pregnancy (78%). Nicotine/tobacco was the most used substance (89% during pregnancy, 89% postpartum) followed by cannabis (53% during pregnancy, 51% following pregnancy), and methamphetamine (48% during pregnancy, 47% following pregnancy).

**Table 3:** Substance use during pregnancies from ages 18–29 among women with past or current substance use disorders (*n* = 98).

**Substance use**	***n***	***%***
Did you use substances during your pregnancy/pregnancies	73	74.5
Did you use substances in the year after your pregnancy/pregnancies?	77	78.6
^a^ **During your pregnancy/pregnancies, which substances did**		
**you use?**		
Alcohol	23	31.5
Nicotine/tobacco	65	89.0
Cannabis	39	53.4
Cocaine	7	9.6
Prescription stimulants not as prescribed	7	9.6
Methamphetamine	35	47.9
Inhalants	1	1.4
Sedatives or sleeping pills	6	8.2
Hallucinogens	1	1.4
Street opioids	16	21.9
Prescription opioids not as prescribed	28	38.4
Other	5	6.8
^a^ **In the year after your pregnancy/pregnancies, which**		
**substances did you use?**		
Alcohol	34	44.2
Nicotine/tobacco	69	89.6
Cannabis	40	51.9
Cocaine	11	14.3
Prescription stimulants not as prescribed	8	10.4
Methamphetamine	36	46.8
Inhalants	5	6.5
Sedatives or sleeping pills	10	13.0
Hallucinogens	6	7.8
Street opioids	18	23.4
Prescription opioids not as prescribed	31	40.3
Other	4	5.2

^a^Among women who indicated use during this period.

### Postpartum challenges among emerging adults

Table 4 summarizes the top 10 challenges during the postpartum period as an emerging adult, when participants felt they needed more support. The major challenge with the highest endorsement of *Yes, Major Challenge*, was “dealing with stress like feeling tense, restless, nervous or anxious, or unable to sleep at night” with 84% indicating this was a major or minor challenge. This was followed by “having the money to pay for the very basics like food, housing, medical care, and heating” (major or minor challenge, 72%), “having someone to trust with intimate thoughts and fears” (major or minor challenge, 71%), and “feeling hopeless or little pleasure doing things” (major or minor challenge, 75%). Supplementary Table S1 provides a complete table summarizing challenges for all respondents.

**Table 4:** Top 10 challenges for women from ages 18–29 with past or current substance use disorders during their most difficult postpartum experience (*n* = 97).

**Challenges**	**Yes, major challenge**		**Yes, minor challenge**		**Not a challenge**	
	***n***	***%***	***n***	***%***	***n***	***%***
Dealing with stress like feeling tense, restless, nervous, or anxious, or is unable to sleep at night	51	52.6	30	30.9	16	16.5
Having the money to pay for the very basics like food, housing, medical care, and heating	44	45.8	25	26.0	27	28.1
Having someone to trust with intimate thoughts and fears	42	43.3	27	27.8	28	28.9
Feeling hopeless or little pleasure doing things	41	42.3	32	33.0	24	24.7
Having reliable transportation to get you to medical appointments, meetings, work, or the things needed for daily living	36	37.1	27	27.8	34	35.1
Finding or keeping work or a job	34	35.1	33	34.0	30	30.9
Feeling like part of a community	31	32.0	38	39.2	28	28.9
Having someone to spend time with and share thoughts and experiences	31	32.0	33	34.0	33	34.0
Having someone to talk to about major life decisions	31	32.0	33	34.0	33	34.0
Finding a stable place to live	30	30.9	27	27.8	40	41.2

One participant did not respond to some of the items. The percentages reported are for observed data. The table is sorted by the challenge with the highest endorsement of “Yes, Major Challenge”.

### Postpartum challenges among women who had a pregnancy loss as an emerging adult

Table 5 summarizes the 10 challenges during the postpartum period as an emerging adult for women who had a pregnancy or infant loss, including miscarriage, neonatal death, or stillbirth (*n* = 44). This was to understand if there were specific needs for women who were grieving as a common, yet overlooked, experience. The top challenges were similar for women who had a pregnancy or infant loss compared to the overall sample, though challenges were more frequently endorsed. The top major challenge was “dealing with stress like feeling tense, restless, nervous or anxious, or unable to sleep at night” (major or minor challenge, 95%) followed by “having the money to pay for the very basics like food, housing, medical care, and heating” (79%), “having someone to trust with intimate thoughts and fears” (major or minor challenge, 77%), and “feeling hopeless or little pleasure doing things” (major or minor challenge, 81%). Additionally, 58% said it was a challenge to access support following their pregnancy loss.

**Table 5:** Top 10 challenges for women from ages 18–29 with past or current substance use disorders for those who experienced pregnancy and infant loss (*n* = 44).

**Challenges**	**Yes, major challenge**		**Yes, minor challenge**		**Not a challenge**	
	***n***	***%***	***n***	***%***	***n***	***%***
Dealing with stress like feeling tense, restless, nervous, or anxious, or is unable to sleep at night	25	58.1	16	37.2	2	4.7
Having the money to pay for the very basics like food, housing, medical care, and heating	22	51.2	12	27.9	9	20.9
Having someone to trust with intimate thoughts and fears	21	48.8	12	27.9	10	23.3
Feeling hopeless or little pleasure doing things	20	46.5	15	34.9	8	18.6
Having reliable transportation to get you to medical appointments, meetings, work, or the things needed for daily living	20	46.5	12	27.9	11	25.6
Finding or keeping work or a job	19	44.2	15	34.9	9	20.9
Getting access to support following a pregnancy loss or termination	18	41.9	7	16.3	18	41.9
Feeling like part of a community	17	39.5	18	41.9	8	18.6
Finding a stable place to live	16	37.2	11	25.6	16	37.2
Finding safety from people who insulted, talk down to you, or screamed or cursed at you	16	37.2	12	27.9	15	34.9

One participant did not respond to some of the items. The percentages reported are for observed data. The table is sorted by the challenge with the highest endorsement of “Yes, Major Challenge”.

Supplementary Table S2 provides a complete table summarizing all challenges for women who experienced pregnancy and infant loss.

### Postpartum challenges among women who had a live birth and retained custody following their pregnancy as an emerging adult

Table 6 summarizes the 10 challenges during the postpartum period as an emerging adult for women who had a live birth and retained custody of their baby after birth (*n* = 69). This was to understand if there were specific needs for women who were caring for a baby. The top challenges were similar for women who had a live birth and custody compared to the overall sample. The top major challenge was “dealing with stress like feeling tense, restless, nervous or anxious, or unable to sleep at night” (major or minor challenge, 81%) followed by “having someone to trust with intimate thoughts and fears” (major or minor challenge, 71%), “having the money to pay for the very basics like food, housing, medical care, and heating” (major or minor challenge, 74%), and “feeling hopeless or little pleasure doing things” (major or minor challenge, 77%).

**Table 6:** Top 10 challenges for women from ages 18–29 with past or current substance use disorders for those who had a live birth and retained custody (*n* = 69).

**Challenges**	**Yes, major challenge**		**Yes, minor challenge**		**Not a challenge**	
	***n***	***%***	***n***	***%***	***n***	***%***
Dealing with stress like feeling tense, restless, nervous, or anxious, or is unable to sleep at night	40	58.8	15	22.1	13	19.1
Having someone to trust with intimate thoughts and fears	34	50.0	14	20.6	20	29.4
Having the money to pay for the very basics like food, housing, medical care, and heating	31	45.6	19	27.9	18	26.5
Feeling hopeless or little pleasure doing things	28	41.2	24	35.3	16	23.5
Having reliable transportation to get you to medical appointments, meetings, work, or the things needed for daily living	26	38.2	21	30.9	21	30.9
Finding or keeping work or a job	25	36.8	25	36.8	18	26.5
Having someone to spend time with and share thoughts and experiences	25	36.8	20	29.4	23	33.8
Having someone to talk to about major life decisions	23	33.8	19	27.9	26	38.2
Finding a stable place to live	22	32.4	19	27.9	27	39.7
Finding a place to live that felt safe (e.g., free of violence)	22	32.4	14	20.6	32	47.1

The table is sorted by the challenge with the highest endorsement of “Yes, Major Challenge”.

Supplementary Table S3 provides a complete table summarizing all challenges for women who retained custody after giving birth.

### Discrimination

Table 7 provides information about discrimination experiences for postpartum emerging adults. The majority of women reported at least one type of discrimination (88%), and most reported discrimination based on their substance use (73%). The second most common reason was income or social class (46%), and the third most common reason was housing status (31%).

**Table 7:** Discrimination experiences for women from ages 18–29 with past or current substance use disorders.

**Discrimination experiences**	***n***	***%***
Your ancestry or national origin	5	5.0
Your gender	16	15.8
Your race	11	10.9
Your age	17	16.8
Your religion	8	7.9
Your height	4	4.0
Your weight	22	21.8
Some other aspect of your physical appearance	18	17.8
Your sexual orientation	6	5.9
Your education or income level	25	24.8
Your housing status	31	30.7
A physical disability	4	4.0
Your shade of skin color	6	5.9
Your affiliation with a tribe	0	0.0
The language you spoke or your accent	0	0.0
Your substance use	74	73.3
Your income or social class	46	45.5
Other	9	8.9
**Total number of discriminations experienced**		
None	12	12.4
1	19	19.6
2	14	14.4
3	14	14.4
4	13	13.4
5	9	9.3
6	10	10.3
7 or more	6	7.2

### Open-ended responses

#### Challenges

In addition to the challenges clarified by the quantitative data, participants provided additional details that further highlighted the complexities of challenges that are not simply resolved. For example, the need for disability supports but not qualifying and the need to earn an income but not wanting to be separated from their child.

“*I wasn't able to work but didn't qualify for social security. Money was tight, and I couldn't afford a vehicle, let alone insurance or gas.”*

“*I received TANF but that was it. They wanted me to work even though my child was 6-months-old. I didn't have anyone I trusted to watch her while I worked, and I wasn't going to leave her with strangers.”*

Participants described fear of trusting people to be supportive because of potential ulterior motives and lack of perceived worthiness.

“*There was absolutely nobody in the world I could trust. Yes, you will help me out, but you have an ulterior motive to continue to help me. Again, why else would you help me. I was unworthy of any special treatment or help.”*

Participants shared about how stigma interfered with engaging in services including fear of judgement.

“*There is a significant amount of stigma surrounding substance abuse and judgement is quickly placed on those struggling. It deterred me from reaching out.”*

“*During recovery, I had a counselor but never felt quite open enough to talk about thoughts and fears and challenges with them.”*

They also reported devastating experiences in loss.

“*The doctor yelled and screamed at me calling me names during the process of having a baby at 25 weeks. After giving birth there was only a Chaplin. I had no family, no help, they wrapped my baby up to die even though he was breathing and had a heartbeat. No one came to help me afterwards for the loss of the child or to assist me with my living child.”*

Participants also described systemic challenges such as not being able to request a new person to work with or not being able to receive needed recovery medications.

“*Child welfare is a broken system. They don't educate you on what you can do when you're dealing with them. You have to do what your social worker says even if she discriminates against you. You are not able to ask for a different social worker. You have to rely on the one person assigned and her biased opinion.”*

“*I needed MAT care after my delivery but the strict laws on Subutex caused me to have to drive to another state to maintain my sobriety for opioid use disorder.”*

#### Opportunities for societal improvements

Participants identified potential solutions to alleviate some of the challenges they experienced. First, participants described the need to reduce stigma, specifically, the need for non-judgmental support that was caring, showed kindness, and included service professionals listening.

“*First of all, LISTEN!! It is so easy to judge someone else, but you'll NEVER fully understand until you've walked a mile in their shoes. I used to say I could never understand how someone could abandon their kids for drugs or any reason at all until my circumstances forced me into a life I could've never imagined. Realize that no one wakes up and decides ‘today I'm going to become a heroin addict' it is almost always some unimaginable trauma that they've gone through and can't cope with that causes them to turn to drugs to escape their feelings. Then they don't know how to stop because it snowballs so quickly. I had a hard time getting help because no one listened. Everyone was so quick to pass judgment and turn me away or look at me like some pathetic loser who didn't deserve to live and that's how I saw myself. I gave up and lost all hope. Addicts are people like you but they're in the dark and they've lost all hope. They need love, understanding and compassion to help bring them back to the light.”*

Participants also described the need for developing community-wide systems of support that better reach youth and families who are experiencing hardship. This included youth prevention, support groups, doulas, childcare, domestic violence services,

“*Gather around our at-risk kids. Our kids being raised by single mothers. Get them involved in formal activities. Make them accountable and own their communities as theirs as well… Give them a family when their original family is too messed up to care at that moment in their life.”*

“*I wish there had been widely known young mom groups that were positive and accepting. There were some places I had, but a place that was open often even just to sit and let the kids play and have people to feel connected to. I think the worst part was feeling lost and disconnected.”*

“*I wish I had a doula or some kind of support… Just told me all of my available rights and options. Have a peer support worker for pregnant mothers as a category in itself. Allow mothers with children exhibiting extreme symptoms to access scans and testing without a pediatrician's referral.”*

“*Having a trusted person to watch my child while I worked, having a stable place to live so I could be with my baby, and money to afford basic stuff. Intervened and offered help and/or resources in order to help support me getting out of the unsafe relationship. That was the root cause of every difficulty I have had in my life since having my kids.”*

“*Offer more support programs, money for diapers not just formula and food. Help with finding stable housing, eviction prevention/rent assistance. Help with transportation/money for gas/repairs. More support for mothers with partners that have substance abuse issues and navigating how to deal with that situation when you don't want to break-up the family, or because you need the other person's help financially.”*

Finally, participants described challenges within the system including the opportunity to improve systems by allowing people to make choices in the service providers that are working with and attention to addressing de-humanizing experiences in corrections settings immediately after childbirth.

“*Give me a new social worker when I ask. Make social workers accountable for what they do and make sure they are treating everyone with fairness and respect. Make the income cutoffs higher so more people could apply for food stamps and childcare.”*

“*After returning from the hospital and spending two days with my baby I was back in jail. The first thing they did was put me in a medical observation cell alone. That might have been good for some women to have that time and space to decompress but I was in excruciating pain. I could not stop crying. If I was awake, I was crying, and I really needed to be around other people and process what I had just experienced in order to begin to move past it. In addition to that, it would have been amazing to have some support for the physical aftermath of the birth regarding the chest pain, etc.”*

#### Strength and resilience

When asked to share about what women were proud of as a postpartum emerging adult living with SUD, participants shared about their recovery, resilience, and personal growth through difficult experiences.

“*With my second pregnancy, first live birth, I had my daughter, and I was able to get clean and sober. I left the hospital with her in my care. No child welfare involvement. Before I was able to reinstate my driver's license or buy a car for myself, I had to walk everywhere with my infant. I walked with her in a stroller across town and back 2–3 times a week for my treatment groups. I learned to walk with a flashlight in my hand against the stroller handle once it started getting dark and I had to walk on the edge of the street due to no sidewalks or bike lanes. I'm very proud of this. I don't think I could persevered through that experience again today. I did what I had to do for myself and my daughter and I succeeded.”*

“*I have basically no emotional support whatsoever. But yet I was strong enough to get an abortion and go through it by myself.”*

Participants reported making profound lifestyle changes upon discovering their pregnancies. Many emphasized their success in maintaining abstinence and engaging in recovery during and after pregnancy, often citing this as a pivotal moment in their recovery journey.

“*I left my kids' dad and that was VERY hard to do! He was very abusive for a long time… So abusive that he punched me so hard that my spleen ruptured, and I had to have emergency surgery… So, I was super proud of myself and I still am!!”*

“*I got my GED a year after my first son. I was 20… just making it out alive, I'm proud of that.”*

“*I have two beautiful children that are my whole world. I wouldn't trade a moment of hardship or suffering because I wouldn't have them today. And my children are the only thing that keeps me hopeful strong and determined to have and provide a better life for myself and my kids.”*

“*I'm not too proud of much. I am proud that I did not make a selfish choice to bring children into this world with a mother that didn't find herself fully till the age of 40. I was supposed to die at 30 yrs old. Instead I had my one and only child, got clean and lived for the first time ever. In the end my child that I held onto saved my life without one little doubt I would've been dead today if not for that boy. Best thing I've ever done. Thank you for letting me participate.”*

Several individuals reflected on how their recovery journeys not only transformed their lives but also allowed them to help others facing similar challenges.

“*Honestly, I survived it. It is the single hardest experience of my life… but I got through it and used that experience to help other people.”*

“*I'm glad I found a MAT doctor who would prescribe me the Subutex I needed and weaned me down so that my son was born healthy without detox. Becoming pregnant against all odds (due to my health) gave me hope because I didn't love myself, but I knew I needed to love and protect the life inside me, and I have maintained my sobriety ever since. I pray that I am able to continue getting the medication I need to treat my opioid use disorder. I now get to help other people know there is hope for them by being a peer support worker.”*

## Discussion

We identified the top challenges for postpartum emerging adults, which were related to anxiety and depressive symptoms, financial struggles, and intimate friendships. We found similar top challenges for individuals who experienced pregnancy and infant loss and for those who were parenting, though those who experienced pregnancy and infant loss more frequently endorsed challenges. Women also reported discrimination based on their substance use, income, and housing status. Women further illuminated challenges, solutions, and strengths through written narratives, highlighting the need for multi-level interventions focused on structural, social, and individual level interventions. We discuss ways to leverage this needs assessment to inform recovery research and service improvements for postpartum emerging adults. In line with the person-centered approach to this study's needs assessment, we invite readers to consider opportunities to think comprehensively about what recovery services could entail.

We found that anxiety symptoms were the top reported challenge among women as they reflected on their experiences. Specifically, most respondents indicated that “dealing with stress like feeling tense, restless, nervous or anxious, or unable to sleep at night” was a challenge. Substance use services, including recovery services, in the United States are often separate from mental health treatment, other medical services, and domestic violence resources (80, 81). This is problematic for numerous reasons including that these are all co-occurring challenges experienced by postpartum emerging adults (30, 52, 54). It is known that there is a need to improve access to mental health treatment among adults with SUD (82) and our data reflect this reality for postpartum emerging adults. Policies in the United States that improve insurance coverage to individuals and for mental health services may contribute to addressing this disparity (83, 84), though there are also mental health service supply gaps that remain a significant challenge (85, 86) and emerging adults experience even further siloed care (87). National and statewide efforts are needed to expand access and improve the quality of mental health care for postpartum emerging adults. Specific to recovery services, these could include things like promoting awareness of mental health services, teaching general anxiety coping skills within recovery community centers, and peer support to address root causes of anxiety.

In addition to improving access to and quality of mental health services, while anxiety and depressive symptoms in part occur because of individual propensity for psychopathology (88, 89), the challenges women described made clear that many of these symptoms are in part or in whole derived from complex, difficult, and traumatic circumstances. These circumstances included inadequate survival resources (e.g., food, housing, transportation), interpersonal violence by partners and systems, societal stigma, and disconnection from loved ones. Thus, anxiety symptoms fueled by contextual factors may require interventions that address those circumstances, rather than supporting individual mental health symptom management alone. For example, related to economic strain, there is evidence that poverty causes anxiety and depression (90). Thus, it was not surprising that “having the money to pay for the very basics like food, housing, medical care, and heating” was the second most endorsed challenge. Antipoverty programs including cash transfers or cash transfers with additional education and advancement incentives improve mental health and improve long term economic stability (90). Implementation and expansion of antipoverty programs is in line with participant reflections that the financial assistance they received was insufficient and unattainable even at wages that did not support meeting their basic needs. Specifically, at times, women risked losing financial assistance if they acquired even a minimum wage job.

In addition to financial strain, social circumstances evoke anxiety or posttraumatic stress symptoms. For example, women who survived intimate partner violence expressed being in a constant state of fear. Many women described social disenfranchisement by medical systems and family members. Structural interventions that promote integrated, de-stigmatized care may facilitate access, improved coordination of care, and recovery outcomes. For example, an educational workshop provided education to direct service providers from siloed intimate partner violence, mental health, and SUD treatment. The workshop included information on understanding co-occurring challenges and bridging silos which decreased service provider stigma and improved their interest in addressing fragmented systems of care (91). These kinds of educational opportunities that incorporate recovery services providers such as recovery housing and recovery community centers may help bridge gaps in knowledge and resource navigation for these organizations.

Pregnancy and infant loss and child removal occurred among more than half of participants. One woman described being verbally abused by her doctor and having her baby die in her arms without medical intervention and others described receiving no support following loss and no education on what to do after a removal. There is limited literature on pregnancy and infant loss among women with SUD. We identified one study which found that among adolescents with high rates of substance use, miscarriage increased the likelihood of suicide attempts across emerging adulthood (92). It is known that pregnancy loss is associated with increased anxiety and depression in the general population (93). Child removal is similarly associated with increased anxiety and depression and increased risk of overdose among birthing people with SUD (94, 95). While grief-sensitive cognitive behavioral therapy and mindfulness interventions are known to improve outcomes for individuals following pregnancy and infant loss (96, 97), we are unaware of any studies that have sought to provide specific supports to women with SUD. This gap in research marks a clear opportunity for intervention during a highly vulnerable period. In addition, this information highlights the needs to consider that postpartum support within recovery services ought to consider the full range of postpartum experiences, including loss and removal. This intersection could be addressed in a training as previously described.

In addition to the needs addressing mental health and economic instability, the need for social connection was apparent in our data. The third most indicated challenge was “having someone to trust with intimate thoughts and fears” and other top challenges included, “feeling like part of a community,” “having someone to spend time with and share thoughts and experiences,” and “having someone to talk to about major life decisions.” One participant reflected that a potential solution was to have a “young mom's group” that could offer informal social connection that was also non-judgmental and well-known. Some interventions have successfully intervened for postpartum adults with SUD through peer services to increase treatment engagement, improve care coordination, and reduce stigma and shame about SUD (98). Others have provided education that included education on improving social relationships to postpartum emerging adults which increased recovery capital, parent empathy, and effective communication with family and intimate partners (99). However, these interventions necessitate formal services involvement. Collaboration with community-based and faith-based organizations, particularly in rural communities that may have fewer available resources otherwise may help facilitate additional informal social supports (100). Expansion of recovery community organizations that include pregnancy, postpartum, and parenting meetups or support groups could be additional strategies to provide social connection for young postpartum people in recovery and prior to recovery.

In addition to our presentation of descriptive survey data, qualitative survey data provided more detailed insights to inform intervention services and research. Specifically, women highlighted that, while they participated in services, it was difficult to learn about all the services that might be available to them. Most women with SUD receive insufficient prenatal care and do not receive any postpartum care (31). Emerging adults also have high unmet SUD treatment needs (101). These gaps in care highlight that postpartum emerging adults may not be interacting with typical service settings, implying a need for creative strategies to help inform them of available services. This could entail embedding pregnancy and postpartum specific peer services in agencies that provide harm reduction services to engage people even before they might be engaged in treatment and recovery services and within recovery services (102). It may also include public campaigns leveraging online and other media advertising (103–105).

Women also described several key strengths that are important to emphasize. Specifically, while women faced obstacles, many also demonstrated notable perseverance. Young postpartum women should never have to endure violence, experience discrimination and shame, or walk in the dark across town with their baby multiple times a week or cross state lines to receive treatment. Despite these societal failings, women kept their children safe from harm, advocated for themselves and their children, and showed up to care appointments. They also attained goals like attending college and learning new skills. The emphasize women placed on attaining these goals is in line with prior research on emerging adults in recovery that describing the significance of traditional normative life stage experiences such as developing relationships, career development, gaining autonomy, and finishing school (22, 23). Moreover, women in our study expressed a deep love for their children and pride in being the best mother that they could be. Affirming and highlighting these strengths and highlighting is an imperative for researchers and practitioners seeking to facilitate positive changes in recovery care (106).

## Limitations and future directions

Our study was not without limitations. First, our sample was predominately White limiting generalizability to other sub-populations who may experience different risk and protective factors stemming from racism and cultural identity, respectively (107–109). We also did not incorporate information on other co-occurring challenges such as physical and intellectual disabilities. Our study was also underpowered to perform statistical analyses to understand sub-group profiles to inform care.

Related to measurement challenges, we could have assessed services utilization and experiences with services which could have more clearly informed current services improvements. Whereas, our study provides information about gaps in support more generally for postpartum emerging adults. It is also possible that some of the questions regarding substance use and challenges do not temporally correspond, though we always provided the framing to participants to consider their most challenging postpartum experience. Related to measuring recovery, this was assessed by whether participants reported abstinence from substances which is an imperfect conceptualization of recovery, though not all individuals in our study identified as abstinent during postpartum. Our data were also collected retrospectively and via self-report which are both limitations and strengths. Specifically, individuals may experience recall bias, but they may also be able to offer a more objective and reflective assessment of their past circumstances. Finally, related to the open-text data we collected, there were opportunities to improve the phrasing of the question about how challenges were experienced by participants. In general, qualitative interviews and observations would also provide more depth to better understand services needs and opportunities, though the present study provides an exploratory step to enhance future research.

## Conclusions

The postpartum period may be an ideal to intervene among emerging adults to address substance use and intersecting challenges. Surveying women in recovery from about their retrospective postpartum experiences during the ages of 18–29, this study aimed to highlight challenges and what individuals would find helpful. Women who participated in this study, the majority of whom were in recovery during postpartum, emphasized the need for improved services for mental health that considers social circumstances, practical resources to address economic and basic needs, and relationship building opportunities. We recommend that research and practice begin to respond this call by expanding access to, integrating, and de-stigmatizing substance use, mental health, medical, and domestic violence care; providing direct financial resources such as cash assistance, housing, gas cards, bus passes, and diapers; and organizing low barrier informal support groups to offer a safe space for connection between young mothers.

## Data Availability

The datasets presented in this article are not readily available. The data may be available by request as long as the request is in line with participant consent for sharing deidentified data. Requests to access the datasets should be directed to ccioffi@uoregon.edu.
