# Supplementary material for: Understanding the needs of postpartum emerging adults with substance use disorders to improve recovery supports
Source: Front Public Health. 2025 Oct 29;13:1521093. doi: 10.3389/fpubh.2025.1521093 (PMC12605474; doi:10.3389/fpubh.2025.1521093)
Supplement: Supplementary file 1 [file Table_1.docx]

**Supplemental Materials**

**Table 1S**

*All challenges for women from ages 18-29 with past or current substance use disorders during their most difficult postpartum experience (n = 97)*

|  | Yes, Major  Challenge | | Yes, Minor Challenge | | Not a Challenge | |
| --- | --- | --- | --- | --- | --- | --- |
|  | *n* | *%1* | *n* | *%* | *n* | *%* |
| Dealing with stress like feeling tense, restless, nervous, or anxious, or is unable to sleep at night | 51 | 52.6 | 30 | 30.9 | 16 | 16.5 |
| Having the money to pay for the very basics like food, housing, medical care, and heating | 44 | 45.8 | 25 | 26.0 | 27 | 28.1 |
| Having someone to trust with intimate thoughts and fears | 42 | 43.3 | 27 | 27.8 | 28 | 28.9 |
| Feeling hopeless or little pleasure doing things | 41 | 42.3 | 32 | 33.0 | 24 | 24.7 |
| Having reliable transportation to get you to medical appointments, meetings, work, or the things needed for daily living | 36 | 37.1 | 27 | 27.8 | 34 | 35.1 |
| Finding or keeping work or a job | 34 | 35.1 | 33 | 34.0 | 30 | 30.9 |
| Feeling like part of a community | 31 | 32.0 | 38 | 39.2 | 28 | 28.9 |
| Having someone to spend time with and share thoughts and experiences | 31 | 32.0 | 33 | 34.0 | 33 | 34.0 |
| Having someone to talk to about major life decisions | 31 | 32.0 | 33 | 34.0 | 33 | 34.0 |
| Finding a stable place to live | 30 | 30.9 | 27 | 27.8 | 40 | 41.2 |
| Finding a place to live that felt safe (e.g., free of violence) | 29 | 29.9 | 25 | 25.8 | 43 | 44.3 |
| Having money to purchase the food you needed | 27 | 27.8 | 36 | 37.1 | 34 | 35.1 |
| Finding safety from people who insulted, talk down to you, or screamed or cursed at you | 27 | 27.8 | 26 | 26.8 | 44 | 45.4 |
| Support with childcare responsibilities | 27 | 27.8 | 22 | 22.7 | 48 | 49.5 |
| Accessing inpatient or residential treatment services | 25 | 25.8 | 15 | 15.5 | 57 | 58.8 |
| Feeling connected to family or people who are like family | 24 | 24.7 | 42 | 43.3 | 31 | 32.0 |
| My medical providers acted like it was my fault that I was impacted by my substance use | 24 | 24.7 | 29 | 29.9 | 44 | 45.4 |
| Concentrating, remembering, or making decisions | 23 | 23.7 | 30 | 30.9 | 44 | 45.4 |
| Because of my substance use, my medical providers were unkind to me | 23 | 23.7 | 24 | 24.7 | 50 | 51.5 |
| Getting access to support following a pregnancy loss or termination | 22 | 22.7 | 11 | 11.3 | 64 | 66.0 |
| Accessing alcohol and drug detox services | 21 | 21.6 | 15 | 15.5 | 61 | 62.9 |
| Accessing a peer support person employed by an organization to help you with recovery | 21 | 21.6 | 19 | 19.6 | 57 | 58.8 |
| Finding safety from people who physically hurt you or threatened you | 21 | 21.6 | 22 | 22.7 | 54 | 55.7 |
| Accessing abstinence-based living environment | 20 | 20.6 | 29 | 29.9 | 48 | 49.5 |
| Attending postpartum care visits | 19 | 19.6 | 16 | 16.5 | 62 | 63.9 |
| Doing things alone like visiting a doctor's office or shopping | 19 | 19.6 | 38 | 39.2 | 40 | 41.2 |
| Accessing outpatient addiction treatment | 19 | 19.6 | 23 | 23.7 | 55 | 56.7 |
| Navigating the child welfare system (e.g., DHS) | 19 | 19.6 | 18 | 18.6 | 60 | 61.9 |
| Because of my substance use, my medical providers seemed uncomfortable with me | 19 | 19.6 | 32 | 33.0 | 46 | 47.4 |
| Finding a place to live where you could meet your basic needs (e.g., working stove, clean water, necessary heat and cooling) | 18 | 18.6 | 17 | 17.5 | 62 | 63.9 |
| Access to a postpartum doula | 18 | 18.6 | 17 | 17.5 | 62 | 63.9 |
| Fear of discrimination prevented accessing needed support | 17 | 17.7 | 19 | 19.8 | 60 | 62.5 |
| Finding a place to live where your health was supported (e.g., good air quality, no mold, no lead paint) | 17 | 17.5 | 20 | 20.6 | 60 | 61.9 |
| Getting the medical care you needed | 16 | 16.5 | 24 | 24.7 | 57 | 58.8 |
| Feeling worthy of receiving adequate and kind care from care providers | 16 | 16.5 | 22 | 22.7 | 59 | 60.8 |
| Finding place to live that felt sanitary (i.e., free of pests) | 15 | 15.5 | 14 | 14.4 | 68 | 70.1 |
| Feeling capable navigating difficult interactions with care providers | 15 | 15.5 | 27 | 27.8 | 55 | 56.7 |
| Having enough food to eat | 15 | 15.5 | 27 | 27.8 | 55 | 56.7 |
| Discrimination prevented you from getting the support you needed | 15 | 15.5 | 16 | 16.5 | 66 | 68.0 |
| Access to education like job training, high school diploma, GED or equivalent | 14 | 14.4 | 19 | 19.6 | 64 | 66.0 |
| Because of my substance use, my medical providers avoided me | 13 | 13.5 | 22 | 22.9 | 61 | 63.5 |
| Getting a postpartum visit with a midwife or OB/GYN scheduled | 13 | 13.4 | 17 | 17.5 | 67 | 69.1 |
| Accessing college recovery programs | 13 | 13.4 | 23 | 23.7 | 61 | 62.9 |
| Accessing state or local recovery community organizations or centers | 13 | 13.4 | 30 | 30.9 | 54 | 55.7 |
| Accessing naloxone or Narcan to reverse overdose | 13 | 13.4 | 12 | 12.4 | 72 | 74.2 |
| Support with knowing how to be a parent | 13 | 13.4 | 25 | 25.8 | 59 | 60.9 |
| Finding a pediatrician who I trusted | 13 | 13.4 | 22 | 22.7 | 62 | 63.9 |
| Accessing 12-step, SMART recovery, or other community-led support groups | 12 | 12.4 | 27 | 27.8 | 58 | 59.8 |
| Access to information about healthy child development | 12 | 12.4 | 14 | 14.4 | 71 | 73.2 |
| Understanding information provided by healthcare providers | 11 | 11.3 | 20 | 20.6 | 66 | 68.0 |
| Support managing breast/chest pain after loss or child removal | 10 | 10.4 | 11 | 11.5 | 75 | 78.1 |
| Accessing a syringe exchange program | 10 | 10.3 | 6 | 6.2 | 81 | 83.5 |
| Accessing medical services in a place that was easy to access for you | 10 | 10.3 | 29 | 29.9 | 58 | 59.8 |
| Support with day-to-day activities such as bathing, preparing meals, shopping, managing finances, etc. | 9 | 9.3 | 29 | 29.9 | 59 | 60.8 |
| Support with milk donation after loss or child removal | 8 | 8.5 | 7 | 7.4 | 79 | 84.0 |
| Support with milk expression after loss or child removal | 8 | 8.2 | 10 | 10.3 | 79 | 81.4 |
| Support meeting your infant feeding goals | 8 | 8.2 | 22 | 22.7 | 67 | 69.1 |
| Support stopping milk production after loss or child removal | 7 | 7.2 | 8 | 8.2 | 82 | 84.5 |
| Finding a specialist to provide support for my child's development | 7 | 7.2 | 13 | 13.4 | 77 | 79.4 |
| Access to a pediatrician for concerns about healthy child development | 6 | 6.2 | 15 | 15.5 | 76 | 78.4 |
| Accessing a pediatrician for child wellness checks | 3 | 3.1 | 12 | 12.4 | 82 | 84.5 |
| Access to the immigration services I or my family needed | 2 | 2.1 | 3 | 3.1 | 92 | 94.8 |

**Table 2S**

*All challenges for women from ages 18-29 with past or current substance use disorders for those who experienced pregnancy and infant loss (n = 44)*

|  | Yes, Major  Challenge | | Yes, Minor Challenge | | Not a Challenge | |
| --- | --- | --- | --- | --- | --- | --- |
|  | *n* | *%* | *n* | *%* | *n* | *%* |
| Dealing with stress like feeling tense, restless, nervous, or anxious, or is unable to sleep at night | 25 | 58.1 | 16 | 37.2 | 2 | 4.7 |
| Having the money to pay for the very basics like food, housing, medical care, and heating | 22 | 51.2 | 12 | 27.9 | 9 | 20.9 |
| Having someone to trust with intimate thoughts and fears | 21 | 48.8 | 12 | 27.9 | 10 | 23.3 |
| Having reliable transportation to get you to medical appointments, meetings, work, or the things needed for daily living | 20 | 46.5 | 12 | 27.9 | 11 | 25.6 |
| Feeling hopeless or little pleasure doing things | 20 | 46.5 | 15 | 34.9 | 8 | 18.6 |
| Finding or keeping work or a job | 19 | 44.2 | 15 | 34.9 | 9 | 20.9 |
| Getting access to support following a pregnancy loss or termination | 18 | 41.9 | 7 | 16.3 | 18 | 41.9 |
| Feeling like part of a community | 17 | 39.5 | 18 | 41.9 | 8 | 18.6 |
| Finding a stable place to live | 16 | 37.2 | 11 | 25.6 | 16 | 37.2 |
| Finding safety from people who insulted, talk down to you, or screamed or cursed at you | 16 | 37.2 | 12 | 27.9 | 15 | 34.9 |
| Finding a place to live that felt safe (e.g., free of violence) | 15 | 34.9 | 14 | 32.6 | 14 | 32.6 |
| Having someone to talk to about major life decisions | 15 | 34.9 | 15 | 34.9 | 13 | 30.2 |
| Concentrating, remembering, or making decisions | 14 | 32.6 | 12 | 27.9 | 17 | 39.5 |
| Having money to purchase the food you needed | 14 | 32.6 | 16 | 37.2 | 13 | 30.2 |
| Because of my substance use, my medical providers were unkind to me | 14 | 32.6 | 10 | 23.3 | 19 | 44.2 |
| My medical providers acted like it was my fault that I was impacted by my substance use | 14 | 32.6 | 12 | 27..9 | 17 | 39.5 |
| Finding a place to live where you could meet your basic needs (e.g., working stove, clean water, necessary heat and cooling) | 13 | 30.2 | 8 | 18.6 | 22 | 51.2 |
| Feeling connected to family or people who are like family | 13 | 30.2 | 20 | 46.5 | 10 | 23.3 |
| Support with childcare responsibilities | 13 | 30.2 | 12 | 27.9 | 18 | 41.9 |
| Finding a place to live where your health was supported (e.g., good air quality, no mold, no lead paint) | 12 | 27.9 | 9 | 20.9 | 22 | 51.2 |
| Accessing abstinence-based living environment | 12 | 27.9 | 13 | 30.2 | 18 | 41.9 |
| Accessing outpatient addiction treatment | 12 | 27.9 | 11 | 25.6 | 20 | 46.5 |
| Having someone to spend time with and share thoughts and experiences | 12 | 27.9 | 21 | 48.8 | 10 | 23.3 |
| Getting the medical care you needed | 11 | 25.6 | 14 | 32.6 | 18 | 41.9 |
| Accessing inpatient or residential treatment services | 11 | 25.6 | 10 | 23.3 | 22 | 51.2 |
| Because of my substance use, my medical providers seemed uncomfortable with me | 11 | 25.6 | 15 | 34.9 | 17 | 39.5 |
| Access to a postpartum doula | 10 | 23.3 | 9 | 20.9 | 24 | 55.8 |
| Feeling worthy of receiving adequate and kind care from care providers | 10 | 23.3 | 10 | 23.3 | 23 | 53.5 |
| Doing things alone like visiting a doctor's office or shopping | 10 | 23.3 | 16 | 37.2 | 17 | 39.5 |
| Accessing alcohol and drug detox services | 10 | 23.3 | 10 | 23.3 | 23 | 53.5 |
| Finding place to live that felt sanitary (i.e., free of pests) | 9 | 20.9 | 8 | 18.6 | 26 | 60.5 |
| Getting a postpartum visit with a midwife or OB/GYN scheduled | 9 | 20.9 | 4 | 9.3 | 30 | 69.8 |
| Attending postpartum care visits | 9 | 20.9 | 6 | 14.0 | 28 | 65.1 |
| Feeling capable navigating difficult interactions with care providers | 9 | 20.9 | 11 | 25.6 | 23 | 53.5 |
| Accessing a peer support person employed by an organization to help you with recovery | 9 | 20.9 | 11 | 25.6 | 23 | 53.5 |
| Having enough food to eat | 9 | 20.9 | 13 | 30.2 | 21 | 48.8 |
| Access to education like job training, high school diploma, GED or equivalent | 9 | 20.9 | 7 | 16.3 | 27 | 62.8 |
| Finding safety from people who physically hurt you or threatened you | 9 | 20.9 | 10 | 23.3 | 24 | 55.8 |
| Navigating the child welfare system (e.g., DHS) | 9 | 20.9 | 9 | 20.9 | 25 | 58.1 |
| Fear of discrimination prevented accessing needed support | 8 | 19.0 | 9 | 21.4 | 25 | 59.5 |
| Accessing state or local recovery community organizations or centers | 8 | 18.6 | 14 | 32.6 | 21 | 48.8 |
| Discrimination prevented you from getting the support you needed | 7 | 16.3 | 8 | 18.6 | 28 | 65.1 |
| Because of my substance use, my medical providers avoided me | 7 | 16.3 | 9 | 20.9 | 27 | 62.8 |
| Support managing breast/chest pain after loss or child removal | 6 | 14.3 | 6 | 14.3 | 30 | 71.4 |
| Accessing 12-step, SMART recovery, or other community-led support groups | 6 | 14.0 | 13 | 30.2 | 24 | 55.8 |
| Accessing medical services in a place that was easy to access for you | 6 | 14.0 | 13 | 30.2 | 24 | 55.8 |
| Support with knowing how to be a parent | 6 | 14.0 | 15 | 34.9 | 22 | 51.2 |
| Support with milk donation after loss or child removal | 5 | 11.9 | 4 | 9.5 | 33 | 78.6 |
| Understanding information provided by healthcare providers | 5 | 11.6 | 15 | 34.9 | 23 | 53.5 |
| Accessing college recovery programs | 5 | 11.6 | 12 | 27.9 | 26 | 60.5 |
| Accessing naloxone or Narcan to reverse overdose | 5 | 11.6 | 5 | 11.6 | 33 | 76.7 |
| Support with milk expression after loss or child removal | 5 | 11.6 | 5 | 11.6 | 33 | 76.7 |
| Access to information about healthy child development | 5 | 11.6 | 9 | 20.9 | 29 | 67.4 |
| Finding a pediatrician who I trusted | 5 | 11.6 | 10 | 23.3 | 28 | 65.1 |
| Support with day-to-day activities such as bathing, preparing meals, shopping, managing finances, etc. | 4 | 9.3 | 16 | 37.2 | 23 | 53.5 |
| Support stopping milk production after loss or child removal | 4 | 9.3 | 3 | 7.0 | 36 | 83.7 |
| Support meeting your infant feeding goals | 4 | 9.3 | 10 | 23.3 | 29 | 67.4 |
| Accessing a syringe exchange program | 3 | 7.0 | 2 | 4.7 | 38 | 88.4 |
| Finding a specialist to provide support for my child's development | 3 | 7.0 | 7 | 16.3 | 33 | 76.7 |
| Access to the immigration services I or my family needed | 2 | 4.7 | 0 | 0.00 | 41 | 95.3 |
| Access to a pediatrician for concerns about healthy child development | 2 | 4.7 | 7 | 16.3 | 34 | 79.1 |
| Accessing a pediatrician for child wellness checks | 1 | 2.3 | 6 | 14.0 | 36 | 83.7 |

**Table 3S**

*All challenges for women from ages 18-29 with past or current substance use disorders for those who had a live birth and retained custody (n = 69)*

|  | Yes, Major  Challenge | | Yes, Minor Challenge | | Not a Challenge | |
| --- | --- | --- | --- | --- | --- | --- |
|  | *n* | *%1* | *n* | *%* | *n* | *%* |
| Dealing with stress like feeling tense, restless, nervous, or anxious, or is unable to sleep at night | 40 | 58.8 | 15 | 22.1 | 13 | 19.1 |
| Having someone to trust with intimate thoughts and fears | 34 | 50.0 | 14 | 20.6 | 20 | 29.4 |
| Having the money to pay for the very basics like food, housing, medical care, and heating | 31 | 45.6 | 19 | 27.9 | 18 | 26.5 |
| Feeling hopeless or little pleasure doing things | 28 | 41.2 | 24 | 35.3 | 16 | 23.5 |
| Having reliable transportation to get you to medical appointments, meetings, work, or the things needed for daily living | 26 | 38.2 | 21 | 30.9 | 21 | 30.9 |
| Finding or keeping work or a job | 25 | 36.8 | 25 | 36.8 | 18 | 26.5 |
| Having someone to spend time with and share thoughts and experiences | 25 | 36.8 | 20 | 29.4 | 23 | 33.8 |
| Having someone to talk to about major life decisions | 23 | 33.8 | 19 | 27.9 | 26 | 38.2 |
| Finding a stable place to live | 22 | 32.4 | 19 | 27.9 | 27 | 39.7 |
| Finding a place to live that felt safe (e.g., free of violence) | 22 | 32.4 | 14 | 20.6 | 32 | 47.1 |
| Feeling like part of a community | 22 | 32.4 | 29 | 42.6 | 17 | 25.0 |
| Finding safety from people who insulted, talk down to you, or screamed or cursed at you | 22 | 32.4 | 15 | 22.1 | 31 | 45.6 |
| Support with childcare responsibilities | 20 | 29.4 | 15 | 22.1 | 33 | 48.5 |
| Accessing inpatient or residential treatment services | 18 | 26.5 | 10 | 14.7 | 40 | 58.8 |
| Having money to purchase the food you needed | 18 | 26.5 | 26 | 38.2 | 24 | 35.3 |
| My medical providers acted like it was my fault that I was impacted by my substance use | 18 | 26.5 | 22 | 32.4 | 28 | 41.2 |
| Concentrating, remembering, or making decisions | 17 | 25.0 | 20 | 29.4 | 31 | 45.6 |
| Because of my substance use, my medical providers were unkind to me | 17 | 25.0 | 19 | 27.9 | 32 | 47.1 |
| Feeling connected to family or people who are like family | 16 | 23.5 | 31 | 45.6 | 21 | 30.9 |
| Finding safety from people who physically hurt you or threatened you | 16 | 23.5 | 13 | 19.1 | 39 | 57.4 |
| Getting access to support following a pregnancy loss or termination | 16 | 23.5 | 8 | 11.8 | 44 | 64.7 |
| Accessing abstinence-based living environment | 14 | 20.6 | 20 | 29.4 | 34 | 50.0 |
| Accessing a peer support person employed by an organization to help you with recovery | 14 | 20.6 | 15 | 22.1 | 39 | 57.4 |
| Because of my substance use, my medical providers seemed uncomfortable with me | 14 | 20.6 | 21 | 30.9 | 33 | 48.5 |
| Finding a place to live where you could meet your basic needs (e.g., working stove, clean water, necessary heat and cooling) | 13 | 19.1 | 12 | 17.6 | 43 | 63.2 |
| Accessing alcohol and drug detox services | 13 | 19.1 | 11 | 16.2 | 44 | 64.7 |
| Navigating the child welfare system (e.g., DHS) | 13 | 19.1 | 11 | 16.2 | 44 | 64.7 |
| Finding a place to live where your health was supported (e.g., good air quality, no mold, no lead paint) | 12 | 17.6 | 15 | 22.1 | 41 | 60.3 |
| Access to a postpartum doula | 12 | 17.6 | 11 | 16.2 | 45 | 66.2 |
| Attending postpartum care visits | 12 | 17.6 | 12 | 17.6 | 44 | 64.7 |
| Fear of discrimination prevented accessing needed support | 11 | 16.4 | 14 | 20.9 | 42 | 62.7 |
| Doing things alone like visiting a doctor's office or shopping | 11 | 16.2 | 27 | 39.7 | 30 | 44.1 |
| Accessing outpatient addiction treatment | 11 | 16.2 | 18 | 26.5 | 39 | 57.4 |
| Discrimination prevented you from getting the support you needed | 11 | 16.2 | 14 | 20.6 | 43 | 63.2 |
| Finding place to live that felt sanitary (i.e., free of pests) | 10 | 14.7 | 10 | 14.7 | 48 | 70.6 |
| Getting the medical care you needed | 10 | 14.7 | 17 | 25.0 | 41 | 60.3 |
| Feeling worthy of receiving adequate and kind care from care providers | 10 | 14.7 | 17 | 25.0 | 41 | 60.3 |
| Having enough food to eat | 10 | 14.7 | 19 | 27.9 | 39 | 57.4 |
| Feeling capable navigating difficult interactions with care providers | 9 | 13.2 | 19 | 27.9 | 40 | 58.8 |
| Because of my substance use, my medical providers avoided me | 9 | 13.2 | 14 | 20.6 | 45 | 66.2 |
| Getting a postpartum visit with a midwife or OB/GYN scheduled | 8 | 11.8 | 10 | 14.7 | 50 | 73.5 |
| Accessing 12-step, SMART recovery, or other community-led support groups | 8 | 11.8 | 15 | 22.1 | 45 | 66.2 |
| Accessing naloxone or Narcan to reverse overdose | 8 | 11.8 | 10 | 14.7 | 50 | 73.5 |
| Access to education like job training, high school diploma, GED or equivalent | 8 | 11.8 | 15 | 22.1 | 45 | 66.2 |
| Support with knowing how to be a parent | 8 | 11.8 | 17 | 25.0 | 43 | 63.2 |
| Finding a pediatrician who I trusted | 8 | 11.8 | 13 | 19.1 | 47 | 69.1 |
| Accessing college recovery programs | 7 | 10.3 | 18 | 26.5 | 43 | 63.2 |
| Accessing state or local recovery community organizations or centers | 7 | 10.3 | 19 | 27.9 | 42 | 61.8 |
| Access to information about healthy child development | 7 | 10.3 | 10 | 14.7 | 51 | 75.0 |
| Support managing breast/chest pain after loss or child removal | 6 | 9.0 | 6 | 9.0 | 55 | 82.1 |
| Accessing medical services in a place that was easy to access for you | 6 | 8.8 | 21 | 30.9 | 41 | 60.3 |
| Support with day-to-day activities such as bathing, preparing meals, shopping, managing finances, etc. | 6 | 8.8 | 20 | 29.4 | 42 | 61.8 |
| Understanding information provided by healthcare providers | 5 | 7.4 | 13 | 19.1 | 50 | 73.5 |
| Support with milk donation after loss or child removal | 4 | 6.0 | 4 | 6.0 | 59 | 88.1 |
| Accessing a syringe exchange program | 4 | 5.9 | 5 | 7.4 | 59 | 86.8 |
| Support with milk expression after loss or child removal | 4 | 5.9 | 6 | 8.8 | 58 | 85.3 |
| Support meeting your infant feeding goals | 4 | 5.9 | 15 | 22.1 | 49 | 72.1 |
| Finding a specialist to provide support for my child's development | 4 | 5.9 | 8 | 11.8 | 56 | 82.4 |
| Support stopping milk production after loss or child removal | 3 | 4.4 | 4 | 5.9 | 61 | 89.7 |
| Access to a pediatrician for concerns about healthy child development | 2 | 2.9 | 9 | 13.2 | 57 | 83.8 |
| Access to the immigration services I or my family needed | 0 | 0.0 | 2 | 2.9 | 66 | 97.1 |
| Accessing a pediatrician for child wellness checks | 0 | 0.0 | 7 | 10.9 | 61 | 89.7 |
